# Successful Outcome of Programmed Death 1 Blockade Plus GemOx for Epstein-Barr Virus-Associated Primary Nodal T/NK Cell Lymphoma: A Case Report

**DOI:** 10.3389/fonc.2021.706865

**Published:** 2021-07-02

**Authors:** Liang Xia, Han-Shuo Zhang, Jing Bao, Yu-Chen Zhao, Hai-Long Xia

**Affiliations:** ^1^ Department of Hematology, The First Affiliated Hospital, Anhui Medical University, Hefei, China; ^2^ Department of Hematology, Chaohu Hospital, Anhui Medical University, Hefei, China

**Keywords:** PD-1 blockade, case report, EBV-associated nodal TNKL, tislelizumab, Gemcitabine and oxaliplatin

## Abstract

Epstein-Barr virus (EBV)-associated lymph nodal T/NK cell lymphoma (nodal TNKL) is a rare and aggressive malignancy with an extremely poor prognosis. Although treatments of extranodal NK/T cell lymphoma are frequently reported, the characteristics and pathogenesis of EBV-associated nodal TNKL are different. However, there is no known effective therapy regimen at present. Here, we reported the clinical efficacy and feasibility of the programmed death 1 (PD-1) blockade therapy regimen in an elderly female patient with EBV-associated nodal TNKL. The patient failed to respond to cyclophosphamide, doxorubicin, vindesine, and prednisone regimen but achieved complete response after three cycles of anti-PD-1 antibody (tislelizumab) combined with gemcitabine and oxaliplatin (GemOx) regimen. The finding indicated that tislelizumab combined with the GemOx regimen may be a potent salvage regimen for EBV-associated nodal TNKL.

## Introduction

Epstein-Barr virus (EBV)-associated lymph nodal T/NK cell lymphoma (nodal TNKL) is a rare aggressive subtype of peripheral T cell lymphoma, not otherwise specified (PTCL-NOS), and belongs to the EBV-associated T/NK cell lymphoproliferative disorder category (1). Unlike extranodal NK/T cell lymphoma (ENKTL), which is characterized by invasion of extranodal tissues and/or organs, nodal TNKL mainly presents with nodal disease (2). Additionally, the CD4^-^/CD8^+^/CD56^-^ phenotype is the main clinical and pathological feature that distinguishes it from the ENKTL (2–4). According to the growth pattern of tumors, the World Health Organization (WHO) 2016 guidelines temporarily classified nodal TNKL as an intranodal type of PTCL-NOS, and EBV-positive (EBV^+^) (5). Due to the lack of effective treatment options for nodal TNKL and its insensitivity to traditional chemotherapy, the median overall survival (OS) of nodal TNKL is extremely poor and significantly worse than that of ENKTL (6). The prognosis of nodal TNKL disease is unsatisfactory, and effective regimens are urgently needed to change the outcome of the disease. Here, we report the case of a patient with nodal TNKL who achieved a successful outcome with a salvage regimen based on a programmed death 1 (PD-1) blockade. The patient agreed to the disclosure of their clinical data.

## Case Description

**Figure 1:**
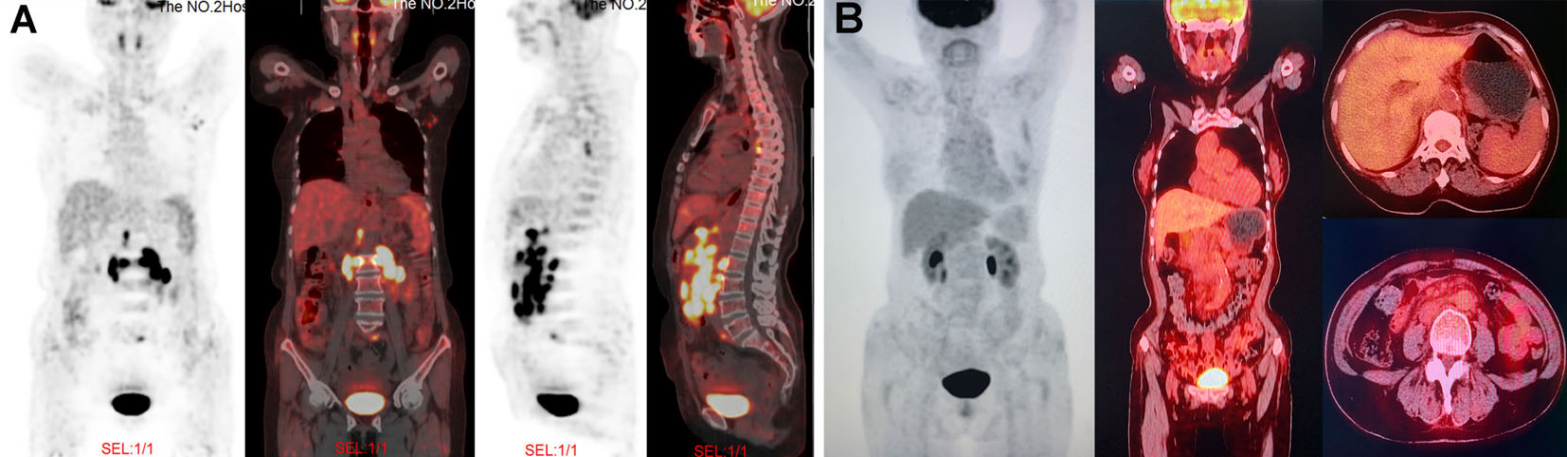
PET/CT images at different time points during treatment. **(A)** At the time of diagnosis, PET/CT revealed the lymphadenopathy in the mediastinum, left axilla, peritoneal retroperitoneal, bilateral diaphragmatic foot, and left peri-iliac vessels; as well as several nodular FDG-avid lesions in the right scapula with hypermetabolic activity (SUVmax: 7.9). **(B)** After four cycles of GemOx plus tislelizumab combination treatment, PET/CT revealed that patient achieved complete response.

**Figure 2:**
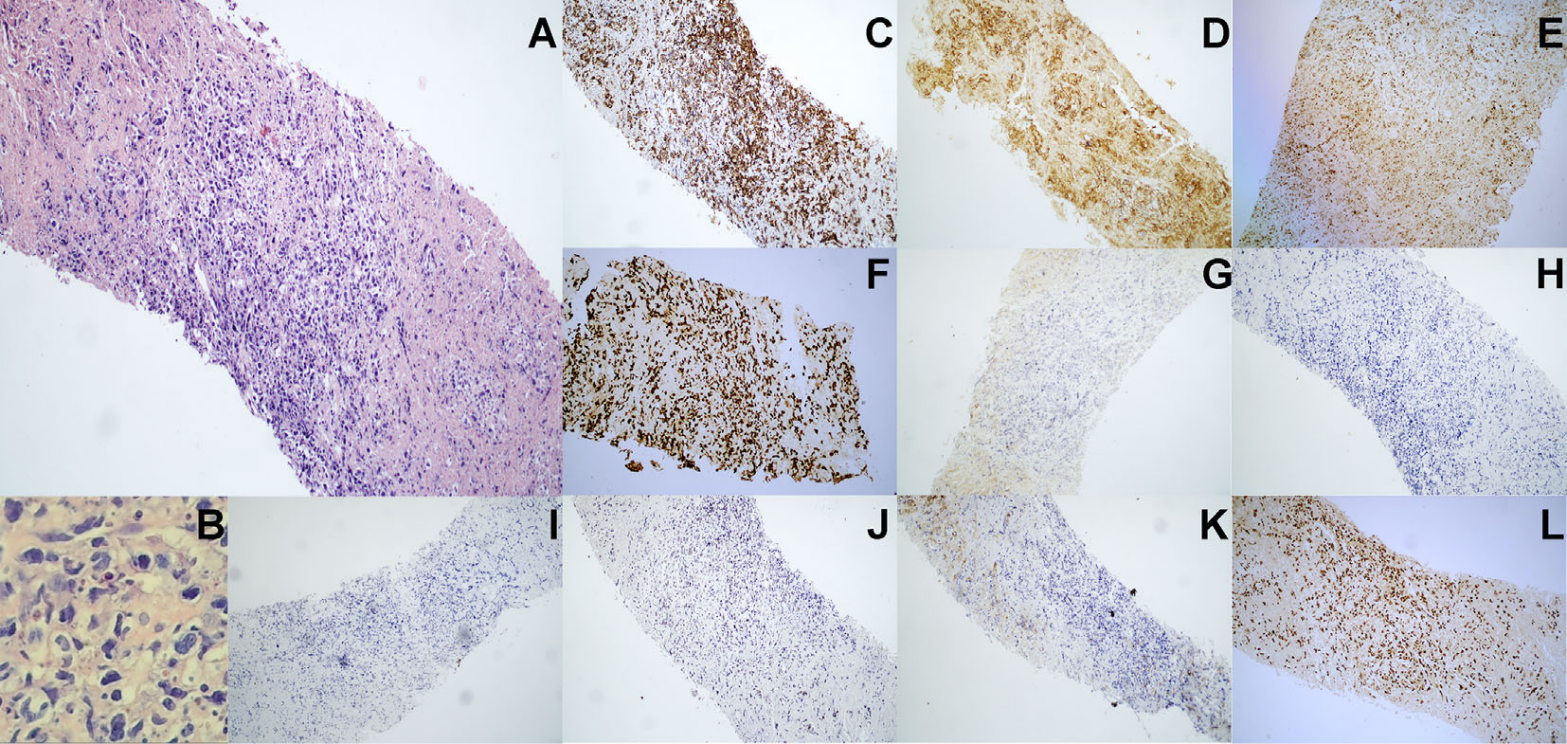
Pathological immunohistochemistry of abdominal lymph node puncture. **(A)** H&E staining revealed the abnormal cell proliferation with lamellar necrosis in the abdominal lymph node biopsy tissue (100× manifestation). **(B)** H&E staining revealed the abnormal cells with large cell volume, abundant cytoplasm, irregular nuclei, thick nuclear chromatin and few visible nucleoli (200× manifestation). **(C–J)** Immunohistochemical staining showed that lymphoma cells were positive for **(C)** CD3, **(D)** CD8, **(E)** TIA-1 and **(F)** Ki-67 (approximately 90%), but were negative for **(G)** CD56, **(H)** CD4, **(I)** CD5, **(J)** CD30 and **(K)** CD20. **(L)** EBV-encoded RNA (EBER) *in situ* hybridization revealed the positive EBV infection.

In August 2020, a 69-year-old Chinese woman with recurrent fever and emaciation for 2 months was diagnosed with stage IIIB nodal TNKL according to the Ann Arbor staging system. Positron emission tomography/computed tomography (PET/CT) revealed the involvement of multiple lymph nodes in both sides of the diaphragm and scapula (**Figure 1A**). Meanwhile, the patient had a 10-year history of hypertension and underwent appendectomy 30 years before TNKL diagnosis and cholecystectomy 2 years prior. Abdominal lymph node biopsy specimen showed large areas of tissue necrosis, focal distribution of abnormal cells with large cell volume, abundant cytoplasm, irregular nuclei, thick nuclear chromatin, and few visible nucleoli under a pathological microscope (**Figures 2A, B**). Immunohistochemical analysis indicated that the tumor cells were positive for CD3, CD8, T-cell intracellular antigen 1 (TIA-1), and granzyme-B (GrB), while were negative for CD4, CD5, CD56, CD30, CD20, and PAX-5 (**Figures 2C–K**). The Ki-67 index was approximately 90% (**Figure 2F**). EBV-encoded RNA (EBER) *in situ* hybridization revealed the positive EBV infection (**Figure 2L**), and a polymerase chain reaction (PCR) detected the plasma EBV-DNA titer of 9.65×10^4^ IU/ml (reference range, <5×10^2^ IU/ml). Bone marrow biopsy did not reveal any evidence of bone marrow invasion. Biochemical examination showed the following: white blood cell: 1.81×10^9/^L (reference range, 3.5-9.55×10^9/^L), neutrophil: 1.43×10^9/^L (reference range, 1.8-6.3×10^9/^L), lymphocyte: 0.18×10^9/^L (reference range, 1.1-3.2×10^9/^L), monocyte: 0.18×10^9/^L (reference range, 0.1-0.6×10^9/^L), hemoglobin: 96 g/L (reference range, 115-150 g/L), platelets: 200×10^9/^L (reference range, 125-350×10^9/^L), and lactate dehydrogenase (LDH): 1956 U/L (reference range, 120-250 U/L). Hemostatic fibrinogen levels were 1.88 g/L (2.0-4.0 g/L), and C-reactive protein levels were 15.42 mg/L (reference range, 0-10 mg/L).

Due to unavailability of vincristine at our institution, the patient was administrated with a modified cyclophosphamide, doxorubicin, vincristine and prednisone (mCHOP) regimen by replacing vincristine with vindesine. After one cycle of mCHOP regimen therapy, the patient’s condition continued to progress, with further aggravated fever, anorexia, and abdominal distension. Imaging showed that the abdominal lymph nodes were larger than they were before chemotherapy. Because of the poor outcome of initial treatment, the patient subsequently received the off-label therapy with the GemOx regimen (gemcitabine: 1 g/m^2^ and oxaliplatin: 100 mg/m^2^; day 1) plus tislelizumab (200 mg; day 2). The combined regimen was administered every 3 weeks. After 3 cycles of therapy, the patient’s symptoms were significantly relieved, and the size of the patient’s largest abdominal lymph nodes decreased from 3×1.7 cm to 1.8×0.5 cm on ultrasound B-scan. The LDH level was reduced to 149 U/L (reference range, 120-250 U/L). We performed PET/CT after 4 cycles of the combined regimen therapy, and the results showed that the tumor had disappeared completely (**Figure 1B**). Then, the patient underwent two more cycles of therapy for consolidation, and the disease remained in complete remission. After 6 cycles of therapy, the patient elected to suspend the treatment. The last follow-up was in March 2021, and the patient was still in good condition without any signs of disease recurrence. During the entire treatment, we did not observe any treatment-related adverse events except for grade 1-2 hematological toxicity (neutropenia) and gastrointestinal symptoms (nausea and retching).

## Discussion

Nodal TNKL develops mostly in the elderly population over 50 years of age and is more common in males and immune-deficient patients with no available treatment options (1). Nodal TNKL is now classified as PTCL-NOS according to the WHO but shows substantial immunophenotypic overlap with ENKTL that arises from cytotoxic T cells. Tumor cells of classical PTCL-NOS originate from helper T (Th) cells, which can be further divided into the TXB21 subtype and GATA3 subtype that originate from Th1 and Th2 cells, respectively, according to gene expression profiling (7). The origin of nodal TNKL is significantly different from that of classic PTCL-NOS, and whether it should be reclassified as a different disease for diagnostic purposes is worthy of further discussion. Nodal TNKL predominantly presents as intra-lymph node lesions, nasal involvement is rare, and the immunophenotype is mainly CD4^-^/CD8^+^/CD56^-^. Nasal involvement is common in ENKTL, but lymph node invasion is rare. The immunophenotype of ENKTL is usually CD4^-^/CD8^-^/CD56^+^. According to gene expression profiling, ENKTL can be divided into three molecular subtypes, namely TSIM, MB, and HEA subtypes. The TSIM subtype mainly involves the activation of the JAK-STAT signaling pathway and the presence of programmed cell-ligand 1 (PD-L1). In the MB subtype, there is a high expression of c-Myc gene (*MYC*) and this subtype is not sensitive to asparaginase. The HEA subtype is mainly characterized by high expression of death domain-associated protein gene (*DAXX*) and is sensitive to histone deacetylase inhibitors (8). A recent study summarized and analyzed the differences in gene expression profiles between nodal TNKL and ENKTL, and found that nodal TNKL genetic features were 14q11.2 deletion, 13q14.3-q21.33 deletion, Xp22.33 deletion, and 1q32.1-q32.3 amplification (9). Besides, nodal TNKL was characterized by upregulation of PD-L1, CD2, CD8, CD3G, CD3D, T cell receptor alpha constant (TRAC) and lymphoid enhancer factor (LEF1), and downregulation of CD56 expression (Ng et al., 2018). The clinical characteristics and immunophenotype of this patient were consistent with typical nodal TNKL. Unfortunately, we were unable to obtain enough tissue for PD-L1 and genetic testing due to the limited number of biopsied tissue samples.

Because the tumor gene expression profile of nodal TNKL is significantly different from that of ENKTL, we predicted that this patient may be less sensitive to asparaginase-based regimens. Meanwhile, in previous research, nodal TNKL was also found to not be sensitive to CHOP-like regimens with poor prognosis and short survival, and the shortest median OS was only 2.47 months (3, 6, 10). This was confirmed by the patient’s first course of treatment. PD-L1 gene amplification and overexpression of PD-L1 are typical features of nodal TNKL and ENKTL. T cell-mediated immunodeficiency may be the main tumor micro-environmental feature of nodal TNKL. T cell receptors bind to the major histocompatibility complex (MHC)-antigen complex on the surface of tumor cells to activate the immune response. When PD-L1 combines with PD-1, T cell function is inhibited and T cells cannot effectively kill tumor cells. PD-1 antibody blocks the binding of PD-1/PD-L1 and restores the activity of T cells, thereby enhancing the immune response (11). A series of encouraging results with PD-1 antibody have been shown in relapsed/refractory (R/R) ENKTL. In a retrospective study of 7 patients with R/R ENKTL that did not respond to L-asparaginase treatment and who then received pembrolizumab 2 mg/kg (every 3 weeks), the objective response rate (ORR) was 100%; and after a median follow-up of 6 months, 5 patients were still in complete remission (CR) (12). Another retrospective study from China included 7 patients with R/R ENKTL who also received pembrolizumab. After a median of 4 courses of treatment, the ORR was 57.1%, including 2 patients who achieved a CR (13). For these reasons, we speculated that the PD-1 antibody may be equally effective for nodal TNKL, although it had not yet been reported. Despite the current patient benefited from this combined regime, the definite efficacy, and tolerability of PD-1 blockade in the treatment for EBV-associated nodal TNKL may not be adequately demonstrated by only one case. Thus, further randomized, multicenter trials evaluating its efficacy as a salvage therapy remain needed.

Although the antibody-dependent cellular phagocytosis (ADCP) of the immunoglobulin G4 (IgG4) antibody is weaker than those of the IgG1 and IgG3 antibodies, it still retains some of the ADCP effect, which may affect the ability of T cells to kill tumor cells, thereby affecting the antitumor efficacy of anti-PD-1 monoclonal antibody treatment (14, 15). The high expression of the macrophage marker CD68 in tumor tissues suggests that there may be abundant macrophages in the tumor micro-environment (6), which may weaken the antitumor effects of traditional PD-1 antibodies. The Fc segment of tislelizumab has been modified to effectively reduce macrophage consumption of T cells, and the ADCP effect is much lower than that of other PD-1 antibodies (16).

Accordingly, we used tislelizumab combined with GemOx (drugs that were not used during the first round of chemotherapy that and are effective for ENKTL) as a salvage treatment and the patient achieved complete remission after 4 cycles. Currently, the incidence of nodal TNKL is extremely low, and it is urgent to carry out multiregional, multicenter clinical trials to explore standard treatment options for these patients. GemOx plus tislelizumab may be a potential effective regimen for nodal TNKL.

## Data Availability Statement

The raw data supporting the conclusions of this article will be made available by the authors, without undue reservation.

## Ethics Statement

The studies involving human participants were reviewed and approved by The First Affiliated Hospital, Anhui Medical University. The patients/participants provided their written informed consent to participate in this study.

## Author Contributions

Conceptualization: H-LX and JB. Methodology: H-LX and JB. Material preparation: LX and H-SZ. Administrative Support: YZ and H-SZ. Formal analysis and data collection: LX and YZ. Data analysis and interpretation: YZ and H-SZ. Writing-original draft preparation: LX and H-SZ. Writing-review and editing: LX and YZ. All authors contributed to the article and approved the submitted version.

## Conflict of Interest

The authors declare that the research was conducted in the absence of any commercial or financial relationships that could be construed as a potential conflict of interest.
